# Outbreak minimization v.s. influence maximization: an optimization framework

**DOI:** 10.1186/s12911-020-01281-0

**Published:** 2020-10-16

**Authors:** Chun-Hung Cheng, Yong-Hong Kuo, Ziye Zhou

**Affiliations:** 1 Logistics and Supply Chain MultiTech R&D Centre Limited, Unit 202, Level 2, Block B, Cyberport 4, 100 Cyberport Road, Hong Kong, Hong Kong, China; 2 Department of Industrial and Manufacturing Systems Engineering, the University of Hong Kong, Pokfulam Road, Hong Kong, China; 3 Department of Systems Engineering and Engineering Management, the Chinese University of Hong Kong, Shatin, New Territories, Hong Kong, China

**Keywords:** Infectious diseases outbreak, COVID-19, SARS, Influence maximization, Optimization, Benders’ decomposition

## Abstract

**Background:**

An effective approach to containing epidemic outbreaks (e.g., COVID-19) is targeted immunization, which involves identifying “super spreaders” who play a key role in spreading disease over human contact networks. The ultimate goal of targeted immunization and other disease control strategies is to minimize the impact of outbreaks. It shares similarity with the famous influence maximization problem studied in the field of social network analysis, whose objective is to identify a group of influential individuals to maximize the influence spread over social networks. This study aims to establish the equivalence of the two problems and develop an effective methodology for targeted immunization through the use of influence maximization.

**Methods:**

We present a concise formulation of the targeted immunization problem and show its equivalence to the influence maximization problem under the framework of the Linear Threshold diffusion model. Thus the influence maximization problem, as well as the targeted immunization problem, can be solved by an optimization approach. A Benders’ decomposition algorithm is developed to solve the optimization problem for effective solutions.

**Results:**

A comprehensive computational study is conducted to evaluate the performance and scalability of the optimization approach on real-world large-scale networks. Computational results show that our proposed approaches achieve more effective solutions compared to existing methods.

**Conclusions:**

We show the equivalence of the outbreak minimization and influence maximization problems and present a concise formulation for the influence maximization problem under the Linear Threshold diffusion model. A tradeoff between computational effectiveness and computational efficiency is illustrated. Our results suggest that the capability of determining the optimal group of individuals for immunization is particularly crucial for the containment of infectious disease outbreaks within a small network. Finally, our proposed methodology not only determines the optimal solutions for target immunization, but can also aid policymakers in determining the right level of immunization coverage.

## Background

The containment of infectious disease outbreaks has been an important issue over decades. In the 21st century, there have still been major epidemics which posed serious global health threats, such as coronavirus disease 2019 (COVID-19), severe acute respiratory syndrome (SARS), dengue fever, middle east respiratory syndrome (MERS), and Ebola virus disease. This study was motivated by a project initiated at the Prince of Wales Hospital (PWH) of Hong Kong [1, 2], a major hospital in the city. The project aimed to investigate solutions for effective and timely responses to possible severe infectious disease outbreaks. PWH suffered from SARS in 2003; there were at least 138 suspected SARS cases potentially acquiring the disease at the facility, where 69 of them were healthcare workers (HCWs) [3]. After SARS, there were reviews of the causes of the hospital outbreak and the effectiveness of the intervention strategies. It was believed that contact tracing was a critical step to identify potential infected cases, as the disease could be spread through person-to-person contact. The recent advancements of information and communication technologies offered a possible and more effective way to establish the contact tracebility, instead of conducting a survey after the outbreak. In the project, a radio-frequency identification (RFID) system was developed to locate individuals (including patients and HCWs) within the facility. While the individuals’ contact activities could be captured through this system, our next question is: what is an effective way to containing the disease? This motivated our current research.

Targeted immunization (TI) is a popular and effective approach to containing epidemic outbreaks. The essence of TI is to identify and immunize at-risk individuals or groups who have higher chances of spreading the disease to a larger population. There are several stages for the containment of infectious disease outbreaks. The very first stage is disease outbreak detection [4–6]. When an outbreak is identified, effective modeling of disease outbreaks and responsive actions for TI would be essential to the containment of the disease spread [7, 8]. The identification of the individuals or groups for immunization aims to mitigate the impacts of the disease spread as far as possible. TI provides protection for not only the targeted individuals but also other members within the same communities, e.g., those who cannot be vaccinated themselves such as infants and pregnant women. When vaccines are scarce with limited budgets, it is especially important to develop effective immunization strategies and to allocate resource optimally for containing infectious disease outbreaks. In the case of healthcare-facility outbreaks of infectious diseases such as SARS and MERS (e.g., [2]), it is essential to protect the healthcare workers (HCWs) who are the frontline medical staff against the outbreak. In this research, we focus on TI for infectious disease outbreaks spread by person-to-person contact [9], where the optimal resource allocation decisions are determined based on the contact network topology.

We consider an equivalent problem which could determine the TI solutions. TI shares similarity with the influence maximization (IM) problem which has been extensively studied in the field of social network analysis. In the IM problem, a user can influence others through social connections, making influence spread over social networks. The IM problem thus is to target a certain number of influential individuals, called “seed” nodes in the social network. These seed nodes are activated at the initial stage, such that the expected influence spread, usually associated with the expected number of nodes that eventually get activated, is maximized. While TI is to identify a set of individuals to minimize the effects of an epidemic spread, the IM problem is to identify a set of individuals to maximize the influence spread. It is natural to see that by considering the population protected from the epidemic outbreak as a reward, target immunization can be transformed to maximizing the reward, which is equivalent to the IM problem [10].

In this work, we first formulate TI as an optimization problem and show that it is equivalent to the standard formulation of the influence maximization problem under the framework of the Linear Threshold (LT) diffusion model. e aim to answer the following research question: can we achieve more effective IT solutions by an optimization approach for the IM problem, as compared with existing methods? To be specific, our research achieves the following contributions:

1. We show that the TI problem is equivalent to the famous IM problem.
2. We provide an explicit and concise formulation of the IM problem under the framework of the LT diffusion model.
3. We develop optimization approaches based on Linear Programming (LP) Relaxation and Bender’s Decomposition.
4. We examine the solutions for the IM problem on real-world large-scale networks and show that the proposed optimization approach achieves more effective solutions, as compared with existing methods.
5. Insights into infectious disease outbreak containment are derived from the computation experiments.

### Related work on the technical tools

We first provide an introduction to the technical tools we adopted in this research – influence maximization, linear threshold model, and Benders’ Decomposition – and review the related work.

#### Influence maximization

The IM problem, originating from the area of viral marketing, was first studied in [11]. Later, an optimization problem was formulated and presented in [12]. After that, their work became the standard approach to solving the IM problem. They proposed an approximate solution based on the greedy algorithm. They also proved that it guarantees a (1−1/*e*−*ε*) bound to the optimal solution for diffusion models with submodular objective functions, such as the Independent Cascade model and the LT model. There are three assumptions in the standard IM model: random activation thresholds, monotonic diffusion functions, and submodular diffusion functions [13]. Mossel and Roch [13] showed that the submodularity holds for the network-level propagation at the global structure if the above three assumptions are satisfied. Soma et al. [14] defined a submodular function on the integer lattice, which extends submodular set functions, and introduced a maximization problem for monotonic submodular function under Knapsack constraints that no longer requires uniform costs. They proposed a polynomial time algorithm with (1−1/*e*) bound to solve the budget allocation problem and compared several strategies for selecting seed sets. Khanna and Lucier [15] proved through bond-percolation-based probabilistic analysis that, on undirected networks, the greedy algorithm could achieve a (1−1/*e*+*c*) bound.

There are two main directions which are extended from Kempe’s work. One is to improve the effectiveness of the solution, as the greedy algorithm gives only (1−1/*e*) approximation to the optimal solution. The other direction is to increase the efficiency of the solution algorithm because the standard solution using Monte Carlo simulations to calculate the expected spread of a seed set requires a significant computation time. However, as far as we know, there is no work in the first direction that aims to improve the effectiveness of the solution. Almost all research work remains in the second direction focusing on speeding up the calculation of expected spread within the framework of a greedy algorithm, e.g., [10, 16–18].

#### Linear threshold model

The IM problem on the LT model is NP-hard [12], and the standard greedy algorithm based on Monte Carlo simulations is computationally expensive. Thus, extensive research has been carried out to advance the performance of approaches to computing the IM process on the LT model. Leskovec et al. [10] proposed a lazy-forward optimization to accelerate the simple greedy algorithm by reducing the number of spread estimation calls, based on the idea that the marginal gain of a node in previous iterations is always larger than (or at least equal to) its marginal gain at the current iteration. Chen et al. [17] proved that calculation of the expected spread on Directed Acyclic Graphs (DAGs) can be completed in linear time. Their algorithm constructs a local DAG for each node. It then iteratively selects a seed using the classic greedy algorithm, which achieves maximum incremental influence spread at each iteration. Goyal et al. [18] proposed an approximation algorithm that utilizes simple paths to calculate the influence for a node and treats the influence for a set as the sum of the influences for all nodes in the set. In this way, the calculation of expected spread is decoupled and becomes additive. Since enumerating all simple paths between a pair of nodes is computationally intractable, they speed up the algorithm by introducing a threshold to prune paths which have little influences.

#### Benders’ decomposition

Benders’ decomposition is a technique in mathematical programming that allows solving some huge mixed integer linear programming (MILP) problems of certain structures. Classical Benders’ decomposition approaches separate a MILP problem into a master problem, usually a MILP problem, and LP subproblems whose dual solutions are used to derive new cuts for the master problem [19]. Hooker and Ottoson [20] proposed Logic-Based Benders’ decomposition where cuts are obtained through the inference dual rather than from the dual formulation of the subproblem. Later, Codato and Fischetti [21] developed and applied Combinatorial Benders’ decomposition, which is a particular case of Logic-Based Benders’ decomposition, to MILP problems involving large numbers of conditional constraints or so-called the big-M constraints. A combinatorial Benders’ cut is derived whenever the solution for the master MILP problem leads an infeasible subproblem. Combinatorial Benders’ decomposition has been successfully applied to various real-world applications such as those related scheduling and assignment problems. Bai [22], for instance, used Combinatorial Benders’ decomposition to solve an optimal allocation problem that tollbooths are allocated to roads for covering the entire road network such that the number of tollbooths required is minimized. By combinatorial decomposition, a large number of logic implications (big-M constraints) can be avoided.

To address the issue that existing IM methods are based on the greedy algorithm, which guarantees only (1−1/*e*) approximation on submodular diffusion functions, we present a novel and concise formulation of the IM problem on the LT model so that it can be solved by more effective optimization techniques. Our approach no longer suffers the limitation of (1−1/*e*) approximation, thus providing solutions with higher quality.

## Methods

We first show the equivalence of the TI problem and the famous IM problem. Then we introduce the LT model and present the proposed Time Aware Influence Maximization (TAIM) model, which takes the temporal nature of influence propagation into the LT model. Notations used in the paper are summarized as follows:

**Table:** 

$G = (\mathcal {V},\mathcal {E})$	=	the graph representing the social network;
*N*	=	number of nodes on the graph, i.e., $|\mathcal {V}|$;
*S*	=	seed set;
*K*	=	number of seed nodes |*S*|;
$\mathcal {N}^{in}(u)$	=	in-neighbor set of node *u*;
$\mathcal {N}^{out}(u)$	=	out-neighbor set of node *u*;
*w*_*u*,*v*_	=	influence weight of node *u* on *v*;
*π*(*S*)	=	expected penalty incurred by *S*;
*π*_*i*_(*S*)	=	penalty incurred by *S* under scenario *i*;
*σ*(*S*)	=	expected number of nodes influenced by *S*;
*σ*_*T*_(*S*)	=	expected number of nodes influenced by *S* within *T* time units;
$\Delta p^{t}_{v}$	=	delta influence of node *v* at time *t*; and
*M*	=	A sufficiently large number.

### Targeted immunization and influence maximization

In the TI problem, a subset of nodes (i.e., individuals) is selected for immunization such that effects of the infectious disease outbreak can be minimized. Let set I represent all possible scenarios of the outbreak. An event *i*∈*I* represent a scenario that starts from a node $s' \in \mathcal {V}$ and spreads through a network $G = (\mathcal {V},\mathcal {E})$. When it reaches a protected node $s \in S \subseteq \mathcal {V}$, the transmission subtree rooted at node *s* is cut off. Thus, a penalty function *π*_*i*_(*s*), dependent on the scenario *i*, is incurred for the population affected before a contaminant reaches the protected node *s*. The affected population is defined as the expected number of people who get infected. The goal of TI is to minimize the expected penalty over all possible scenarios, that is, to minimize the expected number of individuals that would be affected by the outbreak. The TI problem is formulated as:

$$\begin{array}{@{}rcl@{}} \min \qquad \pi(S) &=& \sum_{i\in I} P(i) \pi_{i}(S)\\ s.t.\qquad c(S) &\leq& B \end{array} $$

where *P* is a probability distribution over the events, *c*(*S*) is a cost function for set *S*, and *B* is a limited budget which the total cost cannot exceed.

The IM problem is to determine a seed set such that the expected influence spread is maximized. Different choices of seed nodes lead to different influence spreads that are measured by spread scores. Generally, the spread score is a set function *σ* that maps every seed set *S* to a real number *σ*(*S*). This set function *σ* is the objective to maximize in the problem. With this notion of expected influence spread, the IM problem can be formulated as the following optimization problem:

$$\begin{array}{@{}rcl@{}} \max \qquad \sigma(S) = \sum_{i\in I}P(i)\sigma_{i}(S)\\ s.t.\qquad c(S) &\leq& B \end{array} $$

where *B* is a budget which cannot be exceeded for selecting the seeds.

Following the argument in [10], we show the equivalence between the TI problem and the IM problem. In the TI problem, a maximum penalty *π*(*∞*) is set for not protecting any node in scenario *i*. We consider a scenario-specific penalty reduction *σ*_*i*_(*S*)=*π*_*i*_(*∞*)−*π*_*i*_(*S*) instead of the penalty *π*_*i*_(*S*), which can be viewed as a reward for protecting nodes in *S*. Thus the expected penalty reduction

$$\sigma(S) = \sum_{\text{\i} \in I} P(i)\sigma_{i}(S) = \pi(\text{\O}) - \pi(S), $$

describes the expected reward obtained from providing protection for set *S*. Thus the TI problem and the IM problem become equivalent.

### Linear threshold model

The LT model is defined as follows. In an LT influence graph $G = (\mathcal {V},\mathcal {E})$, an arc (*u*,*v*) is assigned weight *w*_*u*,*v*_ if $(u,v)\in \mathcal {E}$, where $\sum _{u\in \mathcal {V}} w_{u,v} \leq 1, \forall v$. In other words, a node *v* is affected by its neighbor *u* with an influence weight *w*_*u*,*v*_. A condition of having the sum of influence weights for all in-neighbors to *v* no more than one is imposed to ensure that such influence is normalized. When a seed set $S\in \mathcal {V}$ is selected, influence originates from *S* and spreads through the network in discrete steps. Each node *v* independently chooses a threshold *λ*_*v*_ uniformly at random from [0,1]. At each time step *t*, an inactive node *v* becomes active at time step *t*+1 if the total weights from its active in-neighbors reaches its threshold *λ*_*v*_, i.e.,

$$\sum_{u\in \mathcal{N}^{in}(v)} b_{u,v} I(u,t)\geq \lambda_{v}, $$

where *I*(*u*,*t*)=1 if *u* is active at time step *t*, otherwise *I*(*u*,*t*)=0. Let *σ*(*S*) denote the expected number of nodes activated by seed set *S* over all *λ*_*v*_ values from uniform distributions. *σ*(*S*) is referred as the influence spread of seed set *S* on network *G* under the LT model, which is the objective to maximize in the model.

### Problem definition

**Fig. 1:**
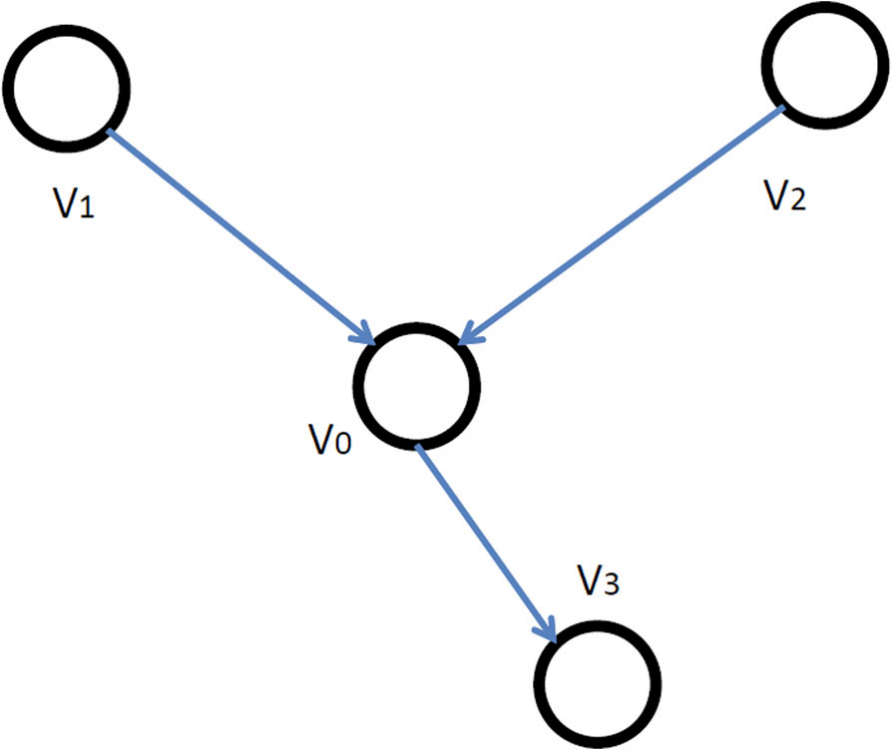
An example of a local network

The standard formulation of the IM problem is general but requires the enumeration of all possible spreading scenarios. Such problem has been shown NP-hard. In this work, we aim to provide a concise formulation to characterize the IM process under the framework of the LT model. To this end, we exploit the discrete propagation nature of the LT model. Consider a local network, e.g., Fig. 1 in which *v*_1_ and *v*_2_ are in-neighbors of *v*_0_ and they are all non-seed nodes. Let *p*^*ti*^ denote the probability that node *v*_*i*_ is active at time *t*. It is obvious that $p_{0}^{t+1}=p_{1}^{t} w_{1,0} + p^{t}_{2} w_{2,0}$ and $p^{t+2}_{0} = p^{t+1}_{1} w_{1,0} + p^{t+1}_{2} w_{2,0}$. Let $\Delta p^{t+1}_{i} = p^{t+1}_{i} - p_{i}^{t}$, then

1 $$\begin{array}{@{}rcl@{}} \Delta p_{0}^{t+1} = \Delta p_{1}^{t} w_{1,0} + \Delta p_{2}^{t} w_{2,0} \end{array} $$

2 $$\begin{array}{@{}rcl@{}} p_{0}^{T} = \sum_{t=1}^{T} \Delta p_{0}^{t} \end{array} $$

where *w*_*u*,*v*_ is influence weight from *u* to *v*. The above equations mean that the influence on a node can be obtained through its delta influence at each time step which is determined by delta influences of its in-neighbors only. We define delta influence as follows.

#### **Definition 1**

(Delta Influence) The delta influence $\Delta p^{t}_{v}$ is the influence increment on node *v* at time *t*, where the influence on *v* means the probability of *v* being activated. The sum of delta influences for a node over time periods [0,*T*] gives the influence on the node at time *T*.

#### **Problem 1**

(Time Aware Influence Maximization Problem) Gvien a directed network $G = (\mathcal {V},\mathcal {E})$ with influence weight *w*_*u*,*v*_∈(0,1] for each arc $(u,v)\in \mathcal {E}$, and a budget *K* restricting the size of seed set, the objective is to determine a seed set $S\in \mathcal {V}$ such that the expected influence within *T* time steps induced by *S*, *σ*_*T*_(*S*), is maximized under the LT model.

### Formulation of time aware influence maximization problem

By Definition 1, we formulate the TAIM problem explicitly as a MILP problem in a concise form:

3 $$\begin{array}{@{}rcl@{}} \max \qquad \sigma(S)=\sum_{i=1}^{N} y_{i} &+& \sum_{t=1}^{T}\sum_{i=1}^{N} x_{i}^{t}  \end{array} $$

4 $$\begin{array}{@{}rcl@{}} s.t. \hspace{1in} \sum_{i=1}^{N} y_{i} &\leq&  \end{array} $$

5 $$\begin{array}{@{}rcl@{}} x^{t}_{i} - M (1-y_{i}) &\leq& 0 \quad \forall i, t \geq 1  \end{array} $$

6 $$\begin{array}{@{}rcl@{}} x^{1}_{i} - \sum_{j \in \mathcal{N}^{in}(i)} w_{ji}y_{j} &\leq& 0 \quad \forall i  \end{array} $$

7 $$\begin{array}{@{}rcl@{}} x^{t}_{i} - \sum_{j \in \mathcal{N}^{in}(i)} w_{ji}x^{t-1}_{j} &\leq& 0 \quad \forall i,t\geq 2  \end{array} $$

8 $$\begin{array}{@{}rcl@{}} y_{i} &\in & \{0,1\} \quad \forall i  \end{array} $$

where $x_{i}^{t} = \Delta _{i}^{t}, i \in \mathcal {V}, t \in \mathcal {T}$. The MILP problem has two sets of decision variables: $\left \{y_{i}: \forall i \in \mathcal {V}\right \}$ and $\left \{x^{t}_{i}: \forall i \in \mathcal {V}, t \in \mathcal {T}\right \}$. $y_{i}, i \in \mathcal {V}$, is binary: 1 if node *i* is selected or 0 otherwise. Continuous variable $x^{t}_{i} := \Delta p_{i}^{t}, i \in \mathcal {V}, t \in \mathcal {T}$, denotes the delta influence of node *i* at time *t*. Objective Function (3) maximizes the expected degree of influence spread initiated by a seed set *S*. That is, the size of the seed set *S* plus the sum of activation probabilities over all nodes and all time periods. Constraint (4) imposes the restriction on the budget. Constraints (5) ensure that the activation probability of any seed node is zero for all time periods. Constraints (6) and (7) establish the relationships among the delta influences of the nodes between consecutive periods according to the LT model.

To solve this MILP problem, a simple approach is proposed to solve its LP Relaxation (and use a heuristic to round LP solutions to generate seed sets that satisfy budget constraint). Another approach to solving the large-scale MILP problem is the Benders’ Decomposition algorithm, which we will elaborate later in this paper. In the experiments, we evaluate solutions obtained from both the LP Relaxation and the Benders’ Decomposition algorithm and compare their performance with popular IM algorithms. The computational experiments suggest that both approaches are effective in solving the TAIM problem, i.e., obtaining high-quality solutions.

### Introduction to Benders’ Decomposition

The original MILP formulation of the TAIM problem is difficult to solve, especially for large-scale instances. Fortunately, for optimization problems in certain forms, Benders’ decomposition techniques, introduced by Benders [19], can be used to obtain an optimal or a near-optimal solution by an iterative procedure. It is an algorithm that decomposes a difficult problem into two manageable parts, the master problem and subproblems. The master problem obtains values for a subset of the variables by solving a relaxed version of the original problem. A subproblem accepts the variables of the master problem and solves for the remaining variables. The subproblem solution is then used to form new constraints or cuts which are added to the master problem and cut off the master problem solution. Master problems and subproblems are solved iteratively in such procedure until no more cuts can be generated. Finally, an optimal solution for the original problem is obtained by combining the solutions of the master problem and subproblem from the last iteration.

The classic Benders’ Decomposition algorithm solves the master problem to optimality at each iteration, which often results in a significant amount of rework and a significant amount of time. In the modern approach, the algorithm solves only a single master MILP problem. Whenever a feasible solution for the master problem is found, it fixes the variables of the master problem to the feasible solution and solves the subproblem. This procedure can be realized using callbacks provided by off-the-shelf MILP solvers such as CPLEX and Gurobi.

### Applying Benders’ Decomposition to TAIM problem

We apply the Benders’ Decomposition algorithm to the TAIM problem, resulting in a master problem and subproblems that are solved iteratively. In this way, part of the complexity of solving the original problem is shifted to two separated simpler problems.

The master problem determines which nodes are selected as seed nodes at the initial stage, the delta influence for each non-seed node at the first period, and the estimate of the delta influence for each node at the remaining periods. Subsequently, the subproblem verifies the estimated delta influence after accepting the solutions to the master problem. Whenever the subproblem identifies an overestimated expectation of the delta influence, an optimality cut is generated and added to the master problem. Since any seed set with size no more than |*S*| is a possible candidate seed set, the subproblem is always feasible whatever a seed set is passed to the master problem, meaning that the subproblem never generates a feasibility cut in this problem. The master problem and subproblems are discussed in detail in the following discussion.

The following MILP problem defines the master problem. Solving the master problem alone leads to a solution that selects seed nodes with the maximum weighted degree. This solution is adopted as the initial solution for the decomposition algorithm, that is the root node of the single solution tree of the master MILP problem. Thus the final solution generated by the decomposition algorithm is at least as good as the heuristic method of choosing the nodes with maximum weighted degrees.

9 $$\begin{array}{@{}rcl@{}} {}\max \sum_{i=1}^{N} \left(y_{i} + x^{1}_{i}\right) + z  \end{array} $$

10 $$\begin{array}{@{}rcl@{}} {}s.t. \hspace{1in} \text{Constraints} (\text{\ref{eq:con1}}), (\text{\ref{eq:con3}}), & \text{and} & (\text{\ref{eq:con5}})\\ {}x^{1}_{i} + My_{i} &\leq& M \quad \forall i  \end{array} $$

11 $$\begin{array}{@{}rcl@{}} {}z&\leq&N\qquad  \end{array} $$

12 $$\begin{array}{@{}rcl@{}} {}\left(y,x^{1},z\right)&\in& \mathcal{O}  \end{array} $$

The master problem has three sets of variables, binary variables $y_{i},i \in \mathcal {V}$ denoting whether node *i* is selected as a seed node, continuous variables $x^{1}_{i},i \in V$ denoting the delta influence of each node at the first period, and an auxiliary continuous variable *z* representing an estimate of the delta influence at remaining periods. Objective Function 9 maximizes the influence spread until the first period and the expected incremental influence afterwards. Constraints (4), (6), and (8) containing only master decision variables become part of the master problem. Constraints (10) are a subset of Constraints (5) for *t*=1. Constraint (11) is used to bound the auxiliary variable *z* to initialize a feasible solution of the master problem. Constraint (12) represents the optimality cuts generated from the subproblems.

The subproblems are defined as follows. Once the master problem has determined the seed nodes that are activated at the initial stage, a subproblem is solved to test whether the expected influence spread in the master problem violates the actual influence spread. That is, the optimality of the solution for this seed set is verified. Whenever an expectation overestimates the actual influence spread, an optimality cut is generated and added to the master problem to correct the estimation. On the other hand, if an expectation is consistent with the actual influence spread, the subproblem determines whether the current feasible integer solution is accepted or not.

13 $$\begin{array}{@{}rcl@{}} {}\max \sum_{t=2}^{T} \sum_{i=1}^{N} x_{i}^{t}  \end{array} $$

14 $$\begin{array}{@{}rcl@{}} s.t. \hspace{1in} x^{2}_{i} &\leq& \sum_{j=1}^{N} w_{ji} x_{j}^{1} \qquad \forall i  \end{array} $$

15 $$\begin{array}{@{}rcl@{}} x^{t}_{i}&\leq& \sum_{j=1}^{N} w_{ji} x_{j}^{t-1} \quad \forall i,t \geq 3  \end{array} $$

16 $$\begin{array}{@{}rcl@{}} x^{t}_{i} &\leq& M(1-y_{i}) \quad \forall i,t\geq 2 \end{array} $$

The continuous variables $x^{t}_{i},i \in \mathcal {V},t \geq 2$ denote the delta influence of node *i* after the second period. Objective Function (13) identifies the actual influence spread at remaining periods. Constraints (14) are formed separately because master variables $x^{1}_{i}, i \in \mathcal {V}$ are now fixed, and Constraints (15) are subsets of Constraints (7), which correspond to *t*=2 and *t*≥3 respectively. Constraint (16) is a subset of Constraints (5) for *t*≥2. Since a subproblem is always feasible, the optimality cut is dependent on only the objective function of the dual subproblem, which is defined in the following form.

17 $$ z\leq \sum_{i=1}^{N} \sum_{j=1}^{N} w_{ji} x_{j}^{1} u_{i} +\sum_{j=2}^{T} \sum_{i=1}^{N} M\left(1-y_{i}\right) u_{(T+t-3)N+i}   $$

where *u* is the optimal solution for the dual problem.

The primal subproblem contains conditional constraints (“big M” coefficients) in Constraints (16), which in general may lead to loose bounds for the master problem due to the weak optimality cuts (12) generated with “big M” coefficients. Here the introduction of “big M” is to impose a constraint such that the subproblem variable $x^{t}_{i} = 0, i \in \mathcal {V},t \geq 2$ only when the corresponding master variable $y_{i},i \in \mathcal {V}$ is 1. In this case, we approximate the exact Benders’ Decomposition by modifying Constraints (16) into the following form:

$$x^{t}_{i} \leq 0 \qquad \forall i \in S, t\geq 2 $$

where *S* is seed set with *y*_*i*_=1 for *i*∈*S*. This means that the conditional constraints are added literally. Another approach to speed up the algorithm is the Combinatorial Benders’ Cuts, which can be generated in addition to the optimality cuts to provide stronger cuts that tighten the master problem.

### Benders’ Decomposition algorithm

The approximate Benders’ Decomposition algorithm for the TAIM problem is presented as follows.

Whenever a candidate solution is found for the master problem during the optimization process, a subproblem is solved after fixing the master variables (*y*^∗^,*x*^1∗^,*z*^∗^) according to this candidate solution. Since the subproblem is always feasible with such master variables, no feasibility cuts will be generated in this decomposition procedure. Instead, optimality cuts are generated and added to the master problem through the following verification. Let *z*_*s*_ denote the optimal objective value of the subproblem and *u* denote the optimal solution for the dual subproblem. If *z*_*s*_<*z*^∗^, meaning that the influence spread is overestimated by the master problem, the optimality cut $z\leq \sum _{i=1}^{N} \sum _{j=1}^{N} w_{ji} x_{j}^{1} u_{i}$ is added to the master problem, and the algorithm continues by solving the master problem again. If *z*_*s*_=*z*^∗^, the solution is accepted. The Benders’ Decomposition algorithm continues by searching for an incumbent solution for the master problem. The solution process ends after the master MILP problem is solved or when a feasible integer solution has been proved to be within a certain optimality gap.

The above algorithm can be modified to seek cuts more aggressively at every node in the master solution tree. Instead of waiting for a new candidate incumbent to add cuts, the algorithm can simply pass a fractional master solution to the subproblem or use a rounded master solution in the subproblem, which may tighten the master problem quickly to prune nodes high in the master search tree.

Algorithm 1 outlines the approximate Benders’ Decomposition algorithm applied to the TAIM problem.


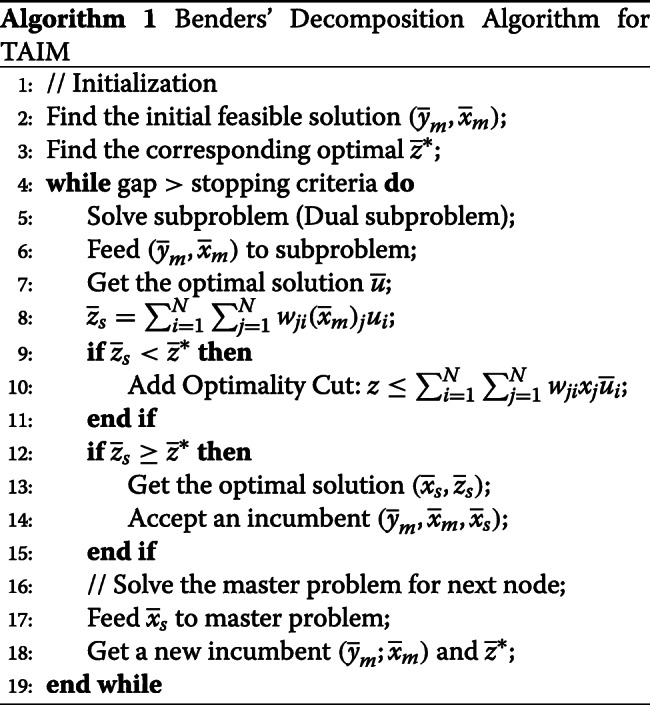


### Computational environment

We conduct a comprehensive computation study to examine the performance of our proposed solution methodology. We present our findings, such as the effectiveness of the methodology, obtained from the computational experiments on three real-world datasets of human networks. The proposed method is implemented using the optimization software CPLEX 12.6. All the experiments are performed on a Linux server running Ubuntu 12.04 with four Intel Xeon CPU E5-2420 processors (1.9GHz) and 193GB memory. The performance of our solution methodology is compared with those resulting from popular IM algorithms and generic heuristic methods.

### Datasets

The characteristics of the datasets are listed in Table 1. Influence weights are obtained by normalizing the original arc weights for incoming arcs of a node, which is similar to the method used in [18]. Specifically, an arc (*u*,*v*) is assigned with a weight *b*(*u*,*v*)=*w*(*u*,*v*)/*W*(*v*), where *w*(*u*,*v*) is the original weight of arc (*u*,*v*) and *W*(*v*) is a normalization factor with $W(v) = \sum _{u\in \mathcal {N}^{in}(v)} w(u,v)$. This assignment of values ensures that the sum of incoming weights for node *v* equals 1.

**Table 1:** Descriptive statistics of the datasets for the computational experiments

**Dataset**	**NetPWH**	**HepCollab**	**SocEpinions**
# of nodes	166	15,233	131,828
# of arcs	3,974	62,796	841,372
Diameter	4	22	14
Average degree	24	4	6
Maximum out-degree	57	64	2,070
Average clustering coefficient	0.7384	0.3137	0.1279
# of components	5	1,781	88,609
# of nodes in largest SCC	64	6,794	41,441
# of arcs in largest SCC	1,792	38,142	693,737

Below provides a brief description of the datasets used in this computational study.

- **NetPWH** A person-to-person contact network of patients and HCWs in two main wards of PWH [1], constructed with the data collected during the project introduced in the “Background” section. The dataset covers activities from December 2011 to March 2012. The network contains 166 nodes, including 56 patients and 110 healthcare workers. Arcs are weighted proportionally to contact frequencies.
- **HepCollab** A collaboration network of scientists on High Energy Physics - Theory section at arXiv.org, from the year 1991 to 2003. The graph contains 15K nodes and 62K arcs. Arcs are weighted based on the number of common papers and the number of authors of the papers.
- **SocEpinions** A who-trust-whom online social network collected from a general consumer review site Epinions.com [23]. An arc indicates whether a member of the site decides to “trust” the other. All the trust relationships interact and form the Web of Trust. The network contains around 132K nodes and 841K arcs.

The computational experiment on the dataset **NetPWH** aims to examine the effectiveness of our proposed methodology in a realistic healthcare facility setting. In particular, we aim to investigate how the person-to-person contact network topology can be integrated into the optimization framework for mitigating the risk of nosocomial diseases outbreaks. To test the scalability of our approach, the two large datasets **HepCollab** and **SocEpinions** are used. The three networks **NetPWH**, **HepCollab**, and **SocEpinions**, respectively, can be considered as small, moderate, and large instances in our computational experiments.

### Algorithms for comparison

We compare our proposed solution methodology with several popular IM algorithms and some generic heuristic methods.

- **Maximum weighted degree** (MAXWEI-DEGREE). Similar to selecting nodes with highest degrees, this heuristic method selects nodes with the *K* highest total out-weights, i.e., $\sum _{v\in \mathcal {N}^{in}(u)} w(u,v)$ for node *u*.
- **Monte-Carlo based cost-effective lazy forward algorithm** (GREEDY). This is a greedy algorithm with CELF optimization proposed in [10, 12]. Monte-Carlo simulations are run to estimate the influence spread of a seed set, and the CELF optimization is to accelerate the spread computation.
- **Local directed acyclic graph** (LDAG). This is the algorithm proposed in [17] that constructs a local DAG for each node to estimate the influence spread. The influence parameter *θ* is set to 1/320, as recommended in [17], to control the size of a local DAG.
- **Simple path algorithm** (SIMPATH). This is the algorithm proposed in [18] that uses simple paths to estimate the influence spread for each node. We set the pruning threshold *η*=10^−3^ and the look-ahead value *l*=4 as recommended by the authors.
- **Solution of LP relaxation with the highest probabilities** (HIGHPROB-LPR). To satisfy the integer constraints, we select *k* nodes that have the highest values in the solution for the LP relaxation. This can be considered as the selection of the nodes with the *K* highest probabilities to be activated at the initial stage.
- **Approximate Benders’ decomposition** (APPROX-BENDERS). This is the approximate Benders’ decomposition algorithm, in which optimality cuts are generated based on the approximate form of the Benders’ subproblem and the conditional constraints are passed to the subproblem literally.
- **Exact solution of LP relaxation** (EXACT-LPR). The seed set is obtained by solving the LP relaxation of the MILP-TAIM problem. Since the binary decision variables are relaxed to continuous values, the solutions obtained by this approach are not feasible but provide an upper bound for the optimization problem. More specifically, the number of seed nodes with non-zero values of the associated decision variables may not be equal to *K*; but the sum of these variables is *K*. The solutions to EXACT-LPR can be used as a benchmark to measure the quality of solution (i.e., proximity of solutions to optimality).

## Results

In this section, we report the computational results and examine the performance of the proposed solution methodology, in terms of computational effectiveness and efficiency. The more detailed insights derived from the computational results will be given in the “Discussion” section.

***Computational effectiveness***

**Fig. 2:**
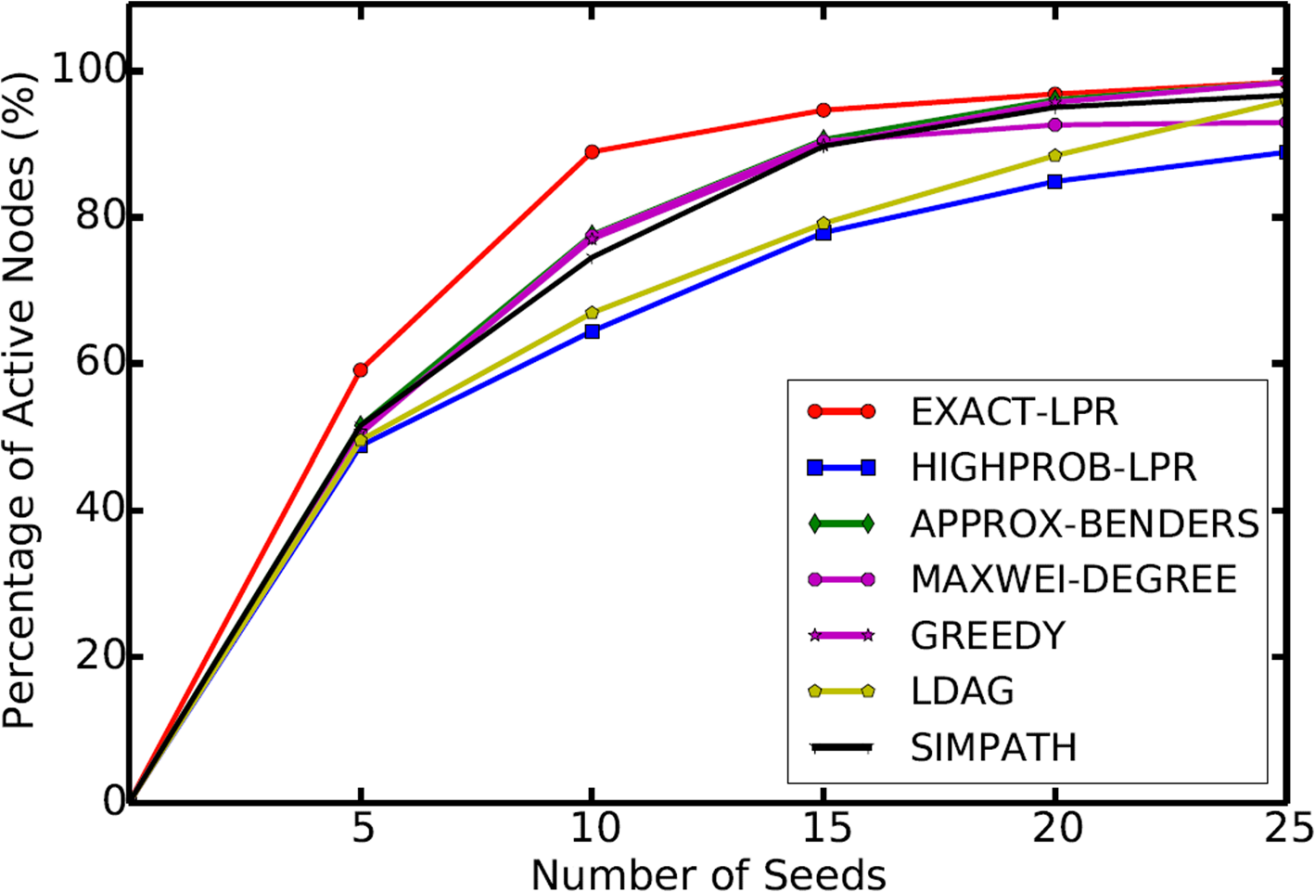
Percentage of active nodes v.s. number of seed nodes on the NetPWH instance

We first evaluate the performance of the algorithms on the dataset **NetPWH**. This experiment can be considered as a test on the effectiveness of the control of an infectious disease outbreak in a healthcare facility setting. This experiment also illustrates the feasibility of utilizing person-to-person contact network topology in the optimization framework for influence maximization, or equivalently, outbreak minimization. Figure 2 shows the percentage of active nodes achieved by different methods against the size of the seed set under the time-aware influence diffusion constraints. The higher the percentage, the more effective an algorithm is. As shown in the figure, APPROX-BENDERS achieves the highest percentage of active nodes for all set sizes. Note that there are gaps between the percentages of active nodes achieved by EXACT-LPR and APPROX-BENDERS. However, as the objective values achieved by EXACT-LPR are upper bounds for the optimal percentages of active nodes, the actual gaps are expected to be smaller than those presented in Fig. 2. While APPROX-BENDERS gives the most effective solutions, the algorithms MAXWEI-DEGREE, GREEDY, and SIMPATH are quite comparable to APPROX-BENDERS. LDAG and HIGHPROB-LPR gave the worst solution effectiveness in this experiment.

**Fig. 3:**
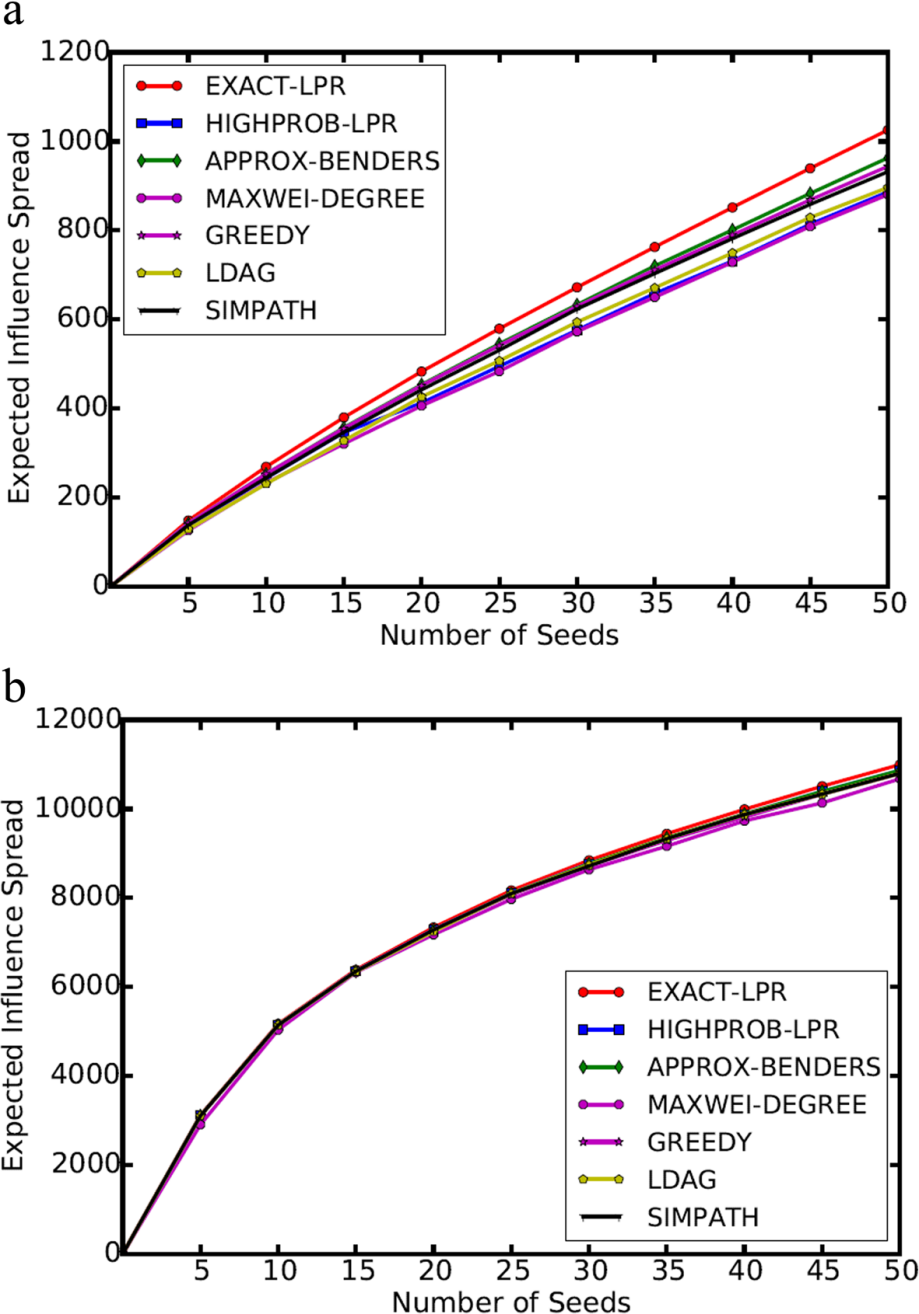
Expected influence spread. **a** HepCollab; **b** SocEpinions

**NetPWH** is a relatively small dataset used to assess the effectiveness of the proposed methodology in a healthcare facility setting. To examine its performance on large-scale datasets, experiments on **HepCollab** and **SocEpinions** are conducted. In this set of experiments, we report the expected influence spread on these large-scale networks, as shown in Fig. 3. We have similar observations as in the experiments on **NetPWH**; EXACT-LPR and APPROX-BENDERS are the two most effective methodologies. However, the differences in the effectiveness of the seed sets become smaller, as compared with those from the experiments on **NetPWH**.

***Computational efficiency and scalability***

**Fig. 4:**
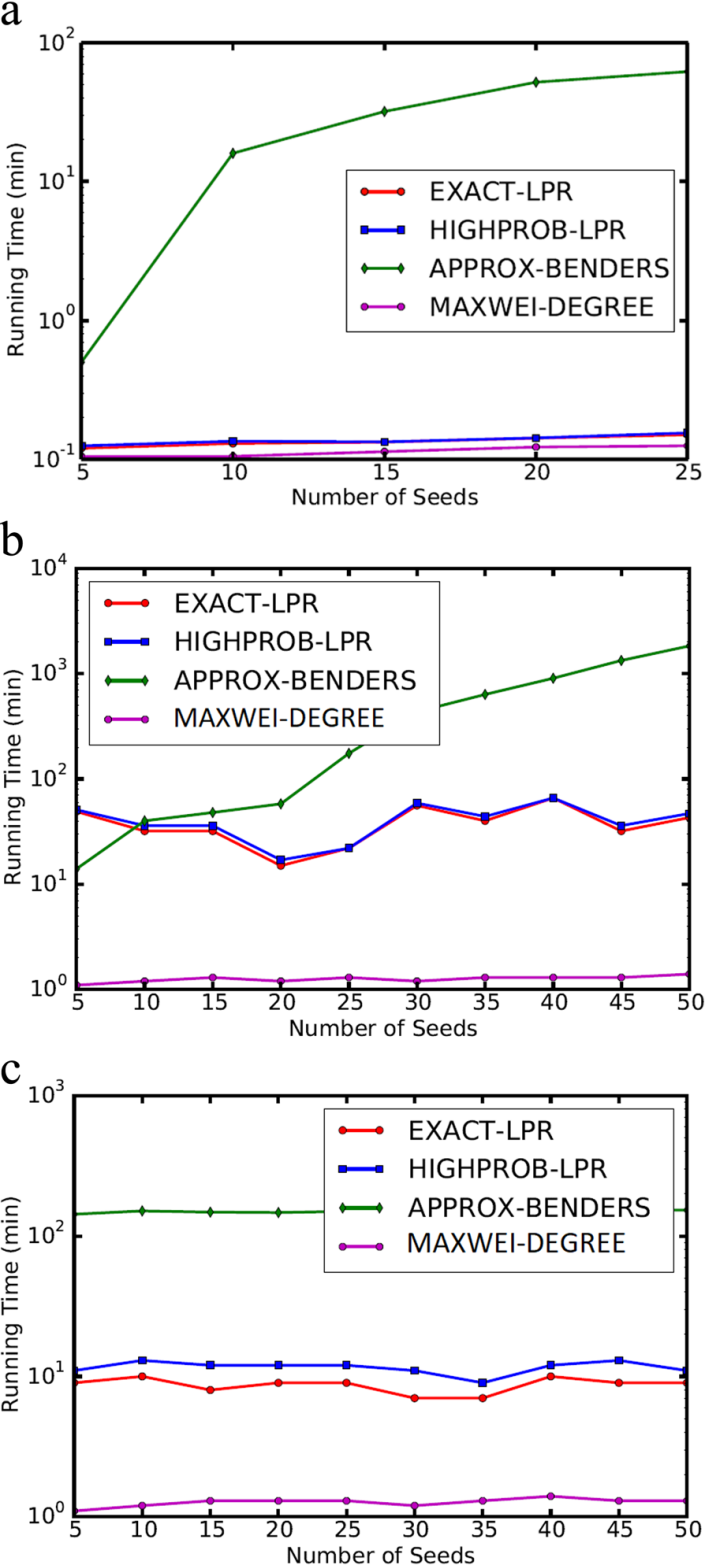
Running times of the algorithms under different environments. **a** NetPWH; **b** HepCollab; **c** SocEpinions

This experiment is to evaluate the efficiency and scalability of our optimization-based approaches – APPROX-BENDERS, EXACT-LPR, and HIGHPROB-LPR – which are expected to be the more computationally expensive. The running time is reported against the size of the seed set on the three datasets, as shown in Fig. 4. For the experiments on **NetPWH** and **HepCollab**, as the size of the seed set increases, the running time of APPROX-BENDERS increases. We believe that it is due to the fact that when the size of the seed set increases, the solution space for the master problem of the Benders’ Decomposition problem increases. Thus, more subproblems have to be solved and more optimality cuts are needed to be generated. On the contrary, EXACT-LPR and HIGHPROB-LPR are rather stable as the size of the seed set is only a parameter in the MILP, which does not increase the problem size. As for the scalability, EXACT-LPR and HIGH-LPR are efficient when dealing with the larger-scale datasets. They finish on the moderate dataset **HepCollab** within 70 min and on the large dataset **SocEpinions** within 10 min to determine the optimal set of 50 seeds. By comparison, APPROX-BENDERS is able to manage the large dataset **SocEpinions**. The computation finish in 110 min for the selection of 50 seeds, while it is not efficient on the moderate dataset **HepCollab**. It completes the experiments on **HepCollab** in around 1000 min for the selection of 30 to 50 seeds. This finding is non-trivial since all methods appear to be more efficient on the large network **SocEpinions** than on the moderate dataset **HepCollab**. The reason is that the experiments are run on a global DAG extracted from **SocEpinions**; however, for **HepCollab**, the algorithms are run on the original network, which contains more loops. We also measure the running time of MAXWEI-DEGREE, which is expected to be highly efficient. In all instances, MAXWEI-DEGREE returns the solution in second.

## Discussion

From the computational experiments, regarding the effectiveness of the seed set identified, we observe that APPROX-BENDERS outperforms other approaches. The quality of the solutions obtained by APPROX-BENDERS is illustrated by the small optimality gaps derived from EXACT-LPR. We observe that the optimality gaps and differences in effectiveness between algorithms are larger on a smaller network, by comparing Figs. 2 with 3. This suggests that an exact method for solving the IM problem is particularly important when dealing with small networks. The rationale is that in a smaller network, an influential node plays a more crucial role in maximizing the influence. In other words, the identification of an optimal group of individuals to immunize is more important for containing outbreaks of infectious diseases in a closed environment, for example, a nosocomial infectious disease outbreak. Thus, the contact frequencies of individuals and the contact network topology would be particularly helpful information in a healthcare facility setting. Hospital administrators may wish to investigate possible solutions for effective contact tracing, e.g., by the adoption of indoor tracking technologies. Existing studies also demonstrate that network topology constructed from surveillance data is useful for the control of disease transmission [24–26]. This study illustrates the feasibility and the significance of utilizing such person-to-person connectivity in the optimization framework for the control of infectious disease outbreaks.

Not surprisingly, there is a tradeoff between computational effectiveness and computational efficiency. A more effective set of seeds requires a more computationally expensive algorithm. In the experiment on the dataset **NetPWH** collected from a hospital, the solution time of the most effective algorithm APPROX-BENDERS is less than 100 minutes. In a practical setting, such solution time is still acceptable. However, for larger-scale instances **HepCollab** and **SocEpinions**, solution times could take almost a day. In cases when quick decisions are needed, heuristics, such as MAXWEI-DEGREE (which requires only to identify individuals of the *K* highest contact frequencies), can be adopted to provide responsive, yet high quality, recommendations for TI.

We also observed that the curves in Figs. 2 and 3 both exhibit concave shapes. This observation is in line with findings from other resource allocation problems; the marginal benefits of adding resources are more significant at a lower resource level. Beyond a certain size of the seeds, the effect of increasing the immunization level becomes mild. Thus, our proposed optimization framework not only identifies optimal solutions for TI, but also helps assess the benefits of expanding the immunization coverage and determine the right immunization level in a cost-effective manner.

## Conclusions

In this work, we study the outbreak minimization problem, which is essential for developing epidemic control strategies. In general, the goal of outbreak control is to minimize the effects of the spread of infectious disease by targeting and preventing “super spreaders” who have significant influences on disease spread over human contact networks. This problem is similar to the famous influence maximization problem studied in social network analysis, which aims to identify a set of influential people to maximize the influence spread through social networks.

Specifically, we show the equivalence of the outbreak minimization and influence maximization problems and present a concise formulation for the influence maximization problem under the LT diffusion model. We then develop optimization approaches based on LP Relaxation and Benders’ Decomposition algorithm, which take into account the contact network topology, to solve the problem. A comprehensive computation study is conducted to evaluate the performance of our proposed solution methodology. Computational results show that the Bender’s Decomposition approach provide more effective solutions for maximizing the influence spread (i.e., minimizing the adverse consequences of infectious disease outbreak).

Our findings suggest that the capability of determining the optimal solutions is particularly important when containing infectious disease outbreaks in smaller networks, e.g., outbreaks of nosocomial infectious diseases. Thus, there is a potential to establish effective contact tracing methods, for example, by indoor tracking technologies, in healthcare facilities and utilize such information for optimal vaccination strategies.

We also illustrate a tradeoff between effectiveness and efficiency of the algorithms. Timely response is key to the success of infectious disease containment. For larger networks which require a long solution time with an exact method, heuristics for good-quality solutions could be a more appropriate alternative to facilitate responsive actions in practice.

Finally, our proposed methodology not only determines the optimal set of individuals for immunization, but also assists the policymakers in assessing the benefits of expanding the immunization coverage and in determining the right immunization level.

## Data Availability

The datasets used and/or analysed during the current study available from the corresponding author on reasonable request.

## Abbreviations

DAG: Directed acyclic graph
HCW: Health care worker
IM: Influence maximization
LP: Linear programming
LT: Linear threshold
MERS: Middle East respiratory syndrome
MILP: Mixed integer linear programming
RFID: Radio-frequency identification
SARS: Severe acute respiratory syndrome
TAIM: Time aware influence maximization
TI: Targeted immunization
